# A Cross-Modal Attention-Driven Multi-Sensor Fusion Method for Semantic Segmentation of Point Clouds

**DOI:** 10.3390/s25082474

**Published:** 2025-04-14

**Authors:** Huisheng Shi, Xin Wang, Jianghong Zhao, Xinnan Hua

**Affiliations:** 1 Department of Remote Sensing Engineering, Henan College of Surveying and Mapping, Zhengzhou 451464, China; shihuisheng@hasm.edu.cn; 2 Beiqi Foton Motor Co., Ltd., Beijing 102206, China; wangxin157@foton.com.cn; 3 School of Geomatics and Urban Spatial Informatics, Beijing University of Civil Engineering and Architecture, Beijing 102627, China; 2108160321009@stu.bucea.edu.cn; 4 State Key Laboratory of Information Engineering in Surveying, Mapping and Remote Sensing, Wuhan University, Wuhan 430000, China

**Keywords:** cross-modal fusion, deep learning, semantic segmentation, point cloud, image, multi-sensor

## Abstract

To bridge the modality gap between camera images and LiDAR point clouds in autonomous driving systems—a critical challenge exacerbated by current fusion methods’ inability to effectively integrate cross-modal features—we propose the Cross-Modal Fusion (CMF) framework. This attention-driven architecture enables hierarchical multi-sensor data fusion, achieving state-of-the-art performance in semantic segmentation tasks.The CMF framework first projects point clouds onto the camera coordinates through the use of perspective projection to provide spatio-depth information for RGB images. Then, a two-stream feature extraction network is proposed to extract features from the two modalities separately, and multilevel fusion of the two modalities is realized by a residual fusion module (RCF) with cross-modal attention. Finally, we design a perceptual alignment loss that integrates cross-entropy with feature matching terms, effectively minimizing the semantic discrepancy between camera and LiDAR representations during fusion. The experimental results based on the SemanticKITTI and nuScenes benchmark datasets demonstrate that the CMF method achieves mean intersection over union (mIoU) scores of 64.2% and 79.3%, respectively, outperforming existing state-of-the-art methods in regard to accuracy and exhibiting enhanced robustness in regard to complex scenarios. The results of the ablation studies further validate that enhancing the feature interaction and fusion capabilities in semantic segmentation models through cross-modal attention and perceptually guided cross-entropy loss (Pgce) is effective in regard to improving segmentation accuracy and robustness.

## 1. Introduction

With the development of deep learning technology, intelligent perception of semantic scenes has been applied in many fields, including autonomous driving, smart cities, robot navigation, and industrial control [1,2,3,4]. Among them, the semantic scene plays a crucial role in autonomous driving, and semantic segmentation is the key technology for sensing the surrounding environment in a driving scene. Deep learning-based semantic segmentation methods can recognize the environment pixel by pixel or point by point, identify and understand important targets around the road, and classify them into different semantic categories. This helps autonomous vehicles to achieve more accurate driving route planning and more intelligent decision making [5,6].

Currently, in-vehicle mobile platforms mainly collect two kinds of data: images and point clouds [7,8]. Images have a rich texture and boundary features, but they are more sensitive to changes in lighting conditions and lack spatial structure information, which may lead to inaccurate semantic segmentation of the scene [9,10]. In comparison, point clouds accurately reflect the spatial structure and can provide accurate 3D coordinates of the surrounding environment, which increases the accuracy and robustness of semantic segmentation and provides more comprehensive and accurate semantic information [11,12,13]. However, point cloud-based semantic segmentation methods face certain challenges due to the sparseness of in-vehicle point clouds, which cannot provide texture information and color descriptions [14,15]. Therefore, it is of great significance to study how to effectively fuse images and point clouds in order to realize in-vehicle point cloud semantic segmentation methods, by taking advantage of their respective strengths and making up for each other’s deficiencies. Despite emerging research [16,17] efforts in regard to multimodal learning for autonomous driving tasks, current approaches exhibit several critical limitations: oversimplified feature fusion strategies, insufficient capabilities in regard to handling dynamic objects and sparse point distributions, and excessive dependence on pre-trained models or multi-stage optimization frameworks. Consequently, the development of advanced in-vehicle point cloud semantic segmentation methods through the use of optimized multimodal fusion remains both technologically significant and scientifically challenging.

Based on the aforementioned issues, we leverage the advantages of point cloud and image data to achieve deep cross-modal feature interaction through the use of cross-modal attention, proposing a cross-modal attention-based multi-sensor feature fusion method (CMF). Unlike existing methods [18,19], we emphasize the importance of fusing RGB image appearance information with point cloud spatial depth information during semantic segmentation. Building on this concept, we propose a perception-oriented multi-sensor fusion scheme that coordinates the fusion of perceptual information from two data modalities in regard to three aspects. First, we employ perspective projection to project the point cloud onto the camera coordinate system, thereby obtaining the spatial depth information corresponding to the RGB image and achieving spatial alignment of the data. Second, we construct a dual-stream feature extractor (SDNet), consisting of a camera stream and a LiDAR stream, which extracts multi-dimensional perceptual features from multimodal sensors. Considering the instability of image information in outdoor environments, we design a residual cross-modal fusion (RCF) module based on cross-modal attention, which integrates the fused features into the LiDAR stream. This not only complements the strengths of point cloud and image data, but also ensures optimal feature fusion between different modalities when subject to dynamic changes. Finally, we propose a perception-guided cross-entropy loss function to measure the perceptual differences between the two data modalities, promoting the reliability of perceptual information fusion.

Our contributions are summarized as follows: (1) We propose a cross-modal attention-driven multi-sensor fusion semantic segmentation method (CMF) for effectively aligning RGB image appearance information and point cloud spatial depth information. (2) We design a dual-stream feature extraction network and achieve multi-level fusion of the two streams through a cross-modal attention-based residual fusion module, enhancing the interaction and fusion of point cloud and image features. (3) We design a perception-guided cross-entropy loss function to measure the perceptual differences between the two modalities, improving the reliability of the fusion results. (4) The relevant experimental results based on the SemanticKITTI and nuScenes benchmark datasets demonstrate the effectiveness of the proposed method.

## 2. Related Work

According to the sensors used, the existing semantic segmentation methods can be broadly divided into three categories: camera-based methods, LiDAR-based methods, and multi-sensor fusion-based methods.

### 2.1. Camera-Based Methods

Camera-acquired images are rich in texture, color, and other information. Camera-based methods achieve semantic segmentation of different targets by assigning specific category labels to each pixel point in the input image. A Full Convolutional Network (FCN) [20], as a landmark network, extends convolutional neural networks to pixel-level semantic segmentation tasks for the first time, and surpasses the traditional semantic segmentation methods in terms of accuracy and efficiency by introducing techniques such as full convolutional architecture and multi-scale feature fusion. On the basis of an FCN, a large number of classical semantic segmentation models have been proposed, such as U-Net [21], SegNet [22], and the DeepLab series [23,24]. Among the semantic segmentation methods for in-vehicle images, DDRNet [25] enhances the information exchange between context and detail branches by introducing bilateral connections, and achieves a balance between accuracy and efficiency. Recently, researchers have introduced a proportional–integral–derivative (PID) controller into convolutional networks and have constructed PIDNet [26], which fuses detail and contextual branches through a boundary attention mechanism and dramatically improves segmentation accuracy at the expense of partial efficiency. Transformer-based methods, such as SETR [27] and SegFormer [28], model the global context through self-attention mechanisms, but they have high computational complexity. Self-supervised learning-based methods like DenseCL [29] improve feature representations through contrastive learning, reducing the reliance on labeled data. However, the method that relies only on the camera is highly affected by the illumination and viewing angle, and lacks stereo information and reliability, which may lead to incomplete segmentation results.

### 2.2. LiDAR-Based Methods

The 3D point clouds acquired by LiDAR technology contain rich spatial structure and geometric information, and have stronger robustness in regard to complex scenes. LIDAR-based methods can be categorized into direct and indirect methods. The direct method uses original point clouds as the input, and the indirect method uses the structurally transformed point clouds as the input. PointNet [30], as a representative of the direct method, first proposes the use of multi-layer perceptrons (MLP) to process point clouds, but ignores the importance of local features. PointNet++ [31], based on PointNet, improves the performance of the network for point cloud segmentation tasks by extracting local features through multiple sampling and grouping. RandLA-Net [32] uses a random sampling method to improve computational efficiency and a local feature aggregation module is utilized to establish local contextual information in order to improve the segmentation efficiency of massive point clouds. Cylinder3D [33] involves a new method for outdoor point cloud segmentation, which utilizes cylindrical segmentation and an asymmetric 3D convolutional network to maintain the 3D geometric structure and properties. In addition, the method includes a correction module based on independent 3D points to reduce the interference of voxel lossy labeling/coding, which leads to excellent segmentation results. SphereFormer [34] aggregates dense near-point information to sparse far points and designs self-concerned radial windows to expand the receptive field, which significantly improves the segmentation accuracy of outdoor scene point clouds. However, direct methods usually involve high computational complexity, which limits their application in the field of autonomous driving. Indirect methods convert 3D point clouds into 2D meshes through projection. This 2D representation allows researchers to investigate efficient network architecture based on existing 2D convolutional networks [35,36]. RangeNet++ [37] is an early representative of the indirect method, which implements 2D convolutional networks based on spherical projections. SalsaNext [38] improves the representation and processing of point clouds by using as inputs the RV maps (range view maps) generated by spherical projections. In a recent study, RangeFormer [39] was used to convert point clouds into 2D representations with long range dependencies, and was trained on low-resolution 2D images through the use of a scalable range view training strategy (STR) to achieve efficient processing and 3D segmentation of point clouds. The Point Transformer [40,41,42] family introduced a transformer layer specifically designed for point clouds, leveraging self-attention to directly extract features from unordered point clouds, without converting them into voxels or grids. However, indirect methods that rely only on point clouds are still insufficient for segmentation when faced with sparse point clouds in the absence of texture information.

### 2.3. Multi-Sensor Fusion-Based Methods

Multi-sensor fusion-based methods improve the accuracy and robustness of 3D point cloud semantic segmentation algorithms by integrating data from camera images and LiDAR point clouds to realize the complementary advantages of both [43,44]. RGBAL [45] converts RGB images into polar grid mapping representations and introduces an early and middle-level fusion strategy. PointPainting [46] enhances the deep learning model’s ability to perceive semantic information by projecting semantic information onto point clouds, thus improving the accuracy of semantic segmentation of point clouds. In regard to PMF [47], point clouds are converted into distance images, so that point clouds and images can achieve multi-level feature fusion based on residual attention in two-dimensional space. Moreover, 2DPASS [16] enhances the representation learning of 3D semantic segmentation networks by distilling multimodal knowledge into a single point cloud modality. In addition, 2D3DNet [17] leverages labeled 2D image data to generate reliable 3D pseudo-labels and, through the use of a multi-view fusion strategy, back-projects these 2D predictions onto 3D point clouds, thereby enabling supervised training of a 3D semantic segmentation model without relying on expensive 3D annotations. Although the aforementioned methods have made some progress in regard to multi-sensor fusion research, they still suffer from insufficient cross-modal feature interaction and use a singular fusion strategy. The CMF framework adopts the collaborative fusion strategy involving multimodal data in the camera coordinate system, as seen in regard to PMF. By integrating camera images and LiDAR point clouds and leveraging a residual fusion module based on cross-modal attention, it achieves a thorough interaction and deep fusion of the two modal features, thereby enhancing the accuracy and robustness of point cloud semantic segmentation.

## 3. Network Principle and Design

In this paper, we propose a cross-modal attention-driven multi-sensor fusion method for the semantic segmentation of point clouds (CMF), aimed at achieving effective cross-modal feature fusion of perceptual information from RGB camera images and LiDAR point clouds. The CMF framework consists of four main parts: (1) data preprocessing, namely point cloud perspective projection transformation; (2) feature extraction, namely a two-stream feature extraction module for both the LiDAR stream and the camera stream; (3) feature fusion, namely a residual fusion module based on cross-modal attention; and (4) a loss function set: a loss function combination innovated with a perceptual guided cross-entropy loss function. The specific process of the CMF framework is as follows: First, the point cloud is projected onto the camera coordinate system via perspective projection. Then, a dual-stream feature extractor is used to extract the perceptual features from both modalities, after which the cross-modal features are fused and injected into the LiDAR stream. Finally, a perception-guided cross-entropy loss function is incorporated into the network optimization.

### 3.1. Dimensional Unification Based on Perspective Projection

The existing dimension unification methods for 3D point clouds with 2D images are mainly categorized into spherical projection and perspective projection. Spherical projection projects the 2D images onto the LiDAR coordinates; however, due to the sparseness of point clouds, the information from the images will be lost to a large extent during this process. To avoid this problem, we use perspective projection to realize the dimensional unification between the 3D point clouds and the 2D images, and project the sparse 3D point clouds onto the camera coordinates to maximize the retention of the pixel information from the images. The specific steps for dimension unification are as follows: first, use the extrinsic matrix to transform the point cloud from the world coordinate system to the camera coordinate system; then, project the point cloud onto the camera’s normalized image plane; and finally, use the intrinsic matrix to convert the normalized coordinates into pixel coordinates.

Let {P,I,L} be one of the training samples from a given dataset, where $P\in R^{4\times N}$ represents a point cloud from LiDAR, N represents the number of points, and 4 represents the four features (x,y,z,r), i.e., the 3D coordinates of each point and a reflectance value r, for each point $P_{i}$ in point cloud P. Let $I\in R^{3\times H\times W}$ be an image from the RGB camera, where H and W represent the height and width of the image, respectively. $L\in R^{N}$ is the set of semantic labels for point cloud P.

In regard to perspective projection, we aim to project the point cloud P from the LiDAR coordinate to the camera coordinate to obtain 2D LiDAR features $\tilde{P}\in R^{C\times H\times W}$. Here, C represents the number of channels of the projected point clouds. Considering the shape of the transformation parameter matrix, the fourth column is added to $P_{i}$ to obtain $P_{i}=(x,y,z,1{)}^{⊤}$, and the projected coordinates ${\tilde{P}}_{i}=(\tilde{x},\tilde{y},\tilde{z}{)}^{⊤}$ of each point cloud are computed in regard to the camera coordinate system.

(1) $$P_{i}=TRP_{i}$$

where $T\in R^{3\times 4}$ is the projection matrix from the LiDAR coordinates in regard to the camera coordinates. $R\in R^{4\times 4}$ is obtained by adding zero-padded augmentation to the rotation matrix $R^{\left(0\right)}\in R^{3\times 3}$ and setting R(4,4)=1. The coordinates (h,w) of the projected point cloud on the image are calculated as follows:

(2) $$h=\tilde{x}/\tilde{z}$$

(3) $$w=\tilde{y}/\tilde{z}$$

where $\tilde{x}$ is the x coordinate of point $P_{i}$ in the camera coordinate system, $\tilde{y}$ is the y coordinate of point $P_{i}$ in the camera coordinate system, and $\tilde{z}$ is the z coordinate in the camera coordinate system, which describes the position of the point in the depth direction, i.e., the distance from the point to the camera. Equations (2) and (3) divided by the z  coordinate take the depth information of the point into account to ensure that the perspective effect of near and far objects is correctly rendered on the image. Specifically, by dividing the x and y coordinates by the z coordinate, normalized coordinates are obtained that take into account the depth information. These normalized coordinates can then be mapped onto the pixel coordinates of the image to determine the position of the point on the image.

### 3.2. CMF Network Structure

**Figure 1:**
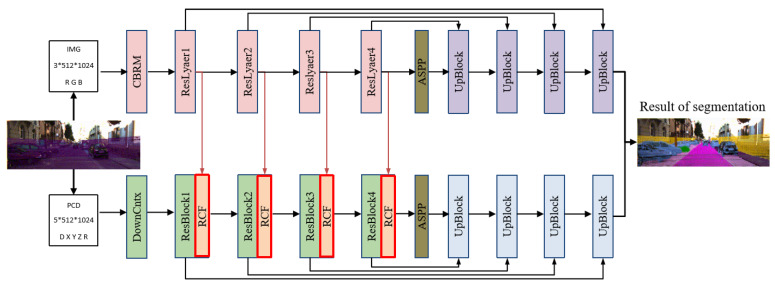
Structure of CMF network.

Since images and point clouds are different modality data, it is difficult for a single network to handle the feature information of both modalities. Inspired by related methods [40,48], we propose a cross-modal fusion-based point cloud segmentation network (CMFNet). The specific structure is shown in Figure 1. The network contains a two-stream feature extractor that processes images from cameras and point clouds from LiDAR, respectively; a cross-modal attention fusion (RCF) module that is used to realize the complementary strengths of image and point cloud features; and a pair of up-sampling modules with cross-layer connectivity, which can gradually restore the high-dimensional feature maps to the size of the input dimensions, respectively.

### 3.3. Residual Fusion Module Based on Cross-Modal Attention

**Figure 2:**
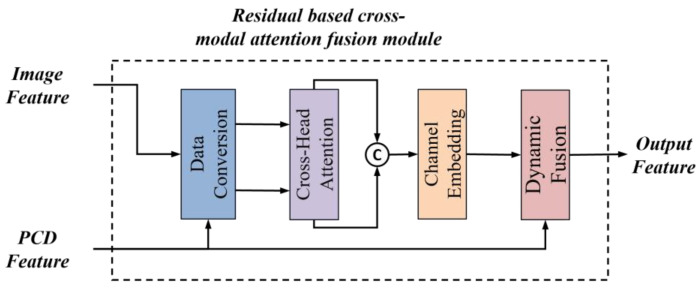
Structure of fusion module.

Since the image features contain details of many objects in the scene and the point cloud features retain more spatial geometric features, we designed a residual fusion module based on cross-modal attention, as shown in Figure 2. The module consists of four submodules: data conversion, the crosspath module, channel embedding, and dynamic fusion. Data conversion projects the features onto a suitable representation space, the crosspath module is responsible for integrating different inputs, the channel embedding submodule is used for in-depth feature extraction, and dynamic fusion is responsible for fusing various types of features. The overall process aims to generate features with higher expressive power that can be used for subsequent tasks, such as classification, target detection, or semantic segmentation. The structure of this module allows the model to better understand the relationships between different data sources when dealing with multimodal data.

**Figure 3:**
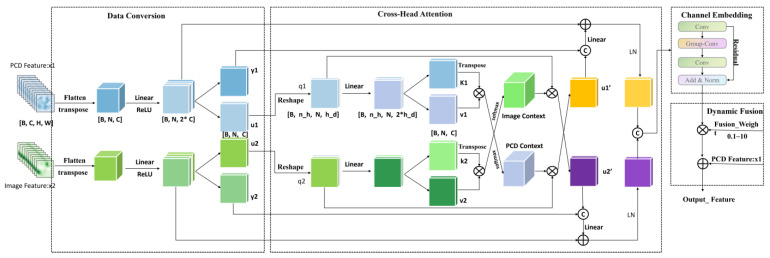
Detailed structure of RCF.

Data conversion consists of two identical channel projection modules that process the input point cloud features $x_{1}$ and image features $x_{2}$, respectively, as shown in Figure 3. The purpose of channel projection is to project the original channel dimensional features onto a lower dimensional space in order to reduce the computational complexity and enhance inter-channel correlation. The cross-attention submodule is used to perform a cross-attention operation between the projected features, $u_{1}$ and $u_{2}$, so that they can “focus” on each other and obtain information about each other. The key steps in the cross-attention computation are as follows: First, we project u_1_ and u_2_ into queries (q) and keys/values (k, v) through linear transformations. Subsequently, the attention mechanism computes the weights between each query and its corresponding keys, enabling targeted information aggregation.

Finally, the attention-weighted values are weighted and summed up using these weights to generate the final cross-attention output. The channel embedding submodule embeds the channels in $v_{1}$ and $v_{2}$ to further change the representation of the channels. The dynamic fusion submodule dynamically assigns weights to the fused features of the channel embedded with the original inputs and then carries out a point-by-point summation fusion. The dynamic weight parameter controls the weights of the fusion and allows the model to learn how to balance the embedded features with the original features.

### 3.4. Perceptual Guided Cross-Entropy Loss Function

Since point clouds are sparse, LiDAR stream networks can only learn the local features of the points, ignoring the shape of the object. In contrast, camera streams are able to capture the shape, color, and texture features of an object from the dense pixels in an image. As a result, there is a large difference between the perceptual features obtained by the camera stream and the LiDAR stream. To address this problem, we introduce a perceptual guided cross-entropy loss function that assists the fusion network in focusing on the fused features from the camera and LiDAR streams. The designed loss function aims to create a consistency and complementarity between the point cloud data and the camera data so as to perform more accurate perceptual tasks.

(4) $$L_{pgce}=L_{pcd}+L_{img}+\alpha \cdot L_{abs}$$

where $L_{pgce}$ represents the perceptual guided cross-entropy loss, $L_{pcd}$ and $L_{img}$ are the perceptual loss terms in terms of the LiDAR stream probability graph and the camera stream probability graph, respectively. Moreover, α represents a dynamic weight, and $L_{abs}$ represents the absolute difference loss term, which represents the absolute difference between the point clouds and images. Where the specific formula for $L_{pcd}$ is as follows:

(5) $$L_{pcd}=CE_{pcd}\cdot W_{pcd}$$

To measure the perceived difference between LiDAR data and camera data, the cross-entropy loss is used to calculate the difference between two probability distributions, $L_{pcd}$ and $L_{img}$, and guide weights are utilized to weigh the point cloud data in regard to the loss calculation based on information importance and a threshold mask. Thus, when the confidence level of the LiDAR data is high, the guide weights are larger and the contribution of the LiDAR data in regard to the loss is more significant. The guideline weights multiply the points with information importance greater than zero by the absolute value of their information importance and weight them according to an adaptive weight function. The weights are then normalized to ensure that their sum is equal to one.

(6) $${\tilde{E}}_{h,w}=-\frac{1}{\log S}\sum_{s=1}^{s}{\tilde{o}}_{s,h,w}\log\left({\tilde{o}}_{s,h,w}\right)$$

where S represents the number of categories in the semantic segmentation and ${\tilde{o}}_{s,h,w}$ represents the probability distribution of the branch outputs. Entropy is used to measure the uncertainty or information content in terms of the probability distribution. Generally, a larger entropy represents a higher uncertainty, i.e., a more unstable probability distribution. We normalize the entropy to $\left.\left(0,1\right)\right.$ using logS. The confidence level is then computed from $\tilde{C}=1-\tilde{E}$. For example, the confidence of the camera stream is higher in terms of the interior of the object, but may be lower at the edges. Therefore, a point cloud confidence threshold mask ${\tilde{\Omega }}_{h,w}$ is created using a predefined threshold τ. These masks are used to determine what information is considered plausible in subsequent calculations.

(7) $${\tilde{\Omega }}_{h,w}=\{\begin{array}{cc} \max\left({\tilde{C}}_{h,w}-C_{h,w},0\right), & \;\mathrm{if}\;{\tilde{C}}_{h,w}>\tau ,\\ 0, & \mathrm{otherwise}. \end{array}$$

(8) $$W_{pcd\left(i\right)}=Im\left(i\right)\cdot \left|Im\left(i\right)\right|\cdot {\tilde{\Omega }}_{h,w}$$

where $Im\left(i\right)$ represents the information importance of the i th data point, which is calculated according to the difference between the confidence level of the LiDAR stream and the camera stream. This value is positive, indicating that the reliability of the LiDAR stream is higher than that of the camera stream. On the contrary, the reliability of the LiDAR stream is lower than that of the camera stream.

(9) $$L=L_{foc}+\lambda L_{lov}+\gamma L_{pgce}$$

where $L_{foc}$, $L_{lov}$, and $L_{pgce}$ represent the Focal Loss [49], the Lovász-Softmax loss [50], and the perceptual guided cross-entropy loss, respectively. Where λ and γ indicate the hyper-parameters that balance different losses.

In addition to the perceptual guided cross-entropy loss, we train the constructed network model using the Focal Loss, a loss function commonly used in existing segmentation work, as well as the Lovász-Softmax loss (Lov loss for short).

## 4. Experimental Results Processing and Analysis

In this section, we will evaluate the performance of the CMF framework based on the SemanticKITTI and nuScenes benchmark datasets. SemanticKITTI is a large-scale dataset based on the KITTI odometry benchmark, featuring semantic annotations for 43,000 scan points, with 21,000 scans (sequences 00–10) available for training and validation. The dataset covers 19 semantic categories for evaluation in semantic benchmarking. The nuScenes dataset contains 1000 driving scenarios in diverse weather and lighting conditions. These scenarios are divided into 28,130 training frames and 6019 validation frames, collected by a Velodyne HDL-32E sensor. Unlike the SemanticKITTI dataset, which only provides front-view camera images, the nuScenes dataset includes data from six cameras, providing multiple viewpoints that can be correlated with LiDAR data.

### 4.1. Implementation Details

Using the PyTorch 1.8.1 framework, we implement the proposed method and use ResNet-34 [50] and SalsaNext as the backbone networks for the LiDAR stream and camera stream, respectively. In regard to the LiDAR stream, we made some adjustments to ResNet-34 to ensure that the number of channels in the input feature maps is (64, 128, 256, 512) during cross-modal fusion. Since we process the point clouds in regard to the camera coordinates, the ASPP module is incorporated into the LiDAR stream network in order to adaptively tune the receptive field. We utilize an existing image classification network backbone and initialize the parameters of ResNet-34 using a pre-trained ImageNet model. During training, we use a hybrid optimization approach to train the branches in the model, i.e., the SGD [51] optimizer for the camera stream and the Adam [52] optimizer for the LiDAR stream. All the experiments were conducted using a system equipped with a GeForce RTX 3090 GPU (24GB VRAM). To reduce memory consumption and accelerate training, we utilized Automatic Mixed Precision (AMP). Through extensive experimentation, the hyperparameters λ and γ in the loss function combination were set to 0.5 and 0.5 for optimal performance. In regard to the SemanticKITTI dataset, we used a batch size of 4, with 40 training epochs, wherein the learning rate started at 0.00025 and decayed to 0. For the nuScenes dataset, a larger batch size of 24 and 150 training epochs were adopted due to the sparser point clouds, with the learning rate initialized at 0.001 and similarly decayed to 0 using cosine scheduling [52].

### 4.2. Comparisons of Benchmark Datasets

#### 4.2.1. Results Based on SemanticKITTI

To evaluate our method based on the SemanticKITTI dataset, we compare the CMF framework to several more established LiDAR methods, including SalsaNext, Cylinder3D, and others. Since the SemanticKITTI dataset only provides images from a forward-looking camera, we project the point clouds onto the perspective view and retain only the points available on the images to construct a subset of the SemanticKITTI dataset. We use sequence 08 for validation and the remaining sequences (00–07 and 09–10) are used as training sets.

As seen in Table 1, the CMF framework exhibits the highest segmentation accuracy for the categories of other vehicle, building, fence, and pole, while it achieves the second-best segmentation accuracy for the categories of motorcycle, road, parking, sidewalk, vegetation, trunk, train, traffic sign, and other categories. In different scenarios in terms of the SemanticKITTI dataset, the CMF framework is able to effectively segment various types of vehicles (e.g., bicycles, motorcycles, trucks, etc.) and different types of roads (e.g., sidewalks, vehicular roads, etc.). In addition, the CMF framework can accurately recognize other kinds of elements, such as parking lots, traffic signs, pedestrians, buildings, and green belts. Compared with point cloud projection-based methods, the CMF framework outperforms RangeNet++, SalsaNext, and SequeezeSegV3 in terms of segmentation accuracy by 13.3%, 5.1%, and 11.2%, respectively. The CMF framework also has a significant advantage in comparison with the voxel-based SPVNAS and the point-based RandLa-Net. In regard to the point cloud semantic segmentation network with LiDAR and camera fusion, the CMF framework has an improved accuracy of 10.0%, 8.3%, and 1.3% compared to PointPainting, RGBAL, and PMF, respectively.

#### 4.2.2. Results Based on nuScenes

To further validate the advanced performance of the CMF framework in terms of the fusion strategy and point cloud semantic segmentation, we conducted model training and evaluation based on the nuScenes dataset. The comparative results with other relevant models are presented in Table 2.

To evaluate our method in regard to more complex scenarios, we compared the CMF framework with state-of-the-art methods based on the nuScenes LiDAR segmentation validation set. The experimental results are shown in Table 2. It is worth noting that the point clouds in the nuScenes dataset are sparser than those in the SemanticKITTI dataset (35k points per frame vs. 125k points per frame), making the 3D segmentation task more challenging. In these conditions, the CMF framework achieved the best performance based on the nuScenes validation set. Specifically, the CMF framework outperformed the best LiDAR-only method, RangeFormer, by 1.2% in regard to the mIoU (mean intersection over union). Compared to the PMF, our CMF framework achieved a 2.4% improvement in the mIoU. These results indicate that the CMF framework can effectively handle challenging segmentation tasks involving extremely sparse point clouds, while further validating the superiority of its feature interaction

### 4.3. Qualitative Evaluation

**Figure 4:**
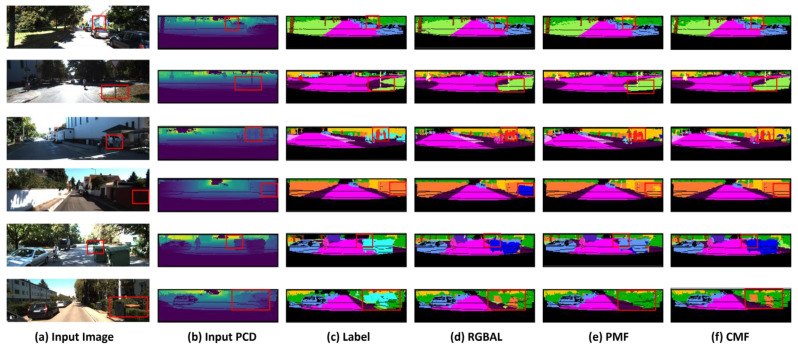
Visualization of RGBAL, PMF, CMF prediction results on based SemanticKITTI dataset.

In order to demonstrate the superiority of the CMF’s segmentation accuracy more intuitively, we visualized the prediction results of the CMF, RGBAL and PMF based on the SemanticKITTI dataset, and used red rectangles to mark the contrasting regions. From Figure 4, we can observe that the CMF framework has a superior segmentation effect at the edge of the object; comparing the fourth row, when the rider is riding a bicycle, the CMF is able to accurately segment the rider and the bicycle compared to RGBAL and PMF. When comparing Figure 4c,e, the cars, traffic signs, and roads, etc., segmented using the CMF have more complete shapes, and the overall scene segmentation effect is more significant. Meanwhile, when comparing Figure 4d,e, the CMF outperforms the PMF in regard to the segmentation of long-distance targets. Therefore, the CMF framework shows better segmentation performance compared to RGBAL and PMF in regard to long distance, interference areas, and small targets.

### 4.4. Distance-Based Evaluation

**Figure 5:**
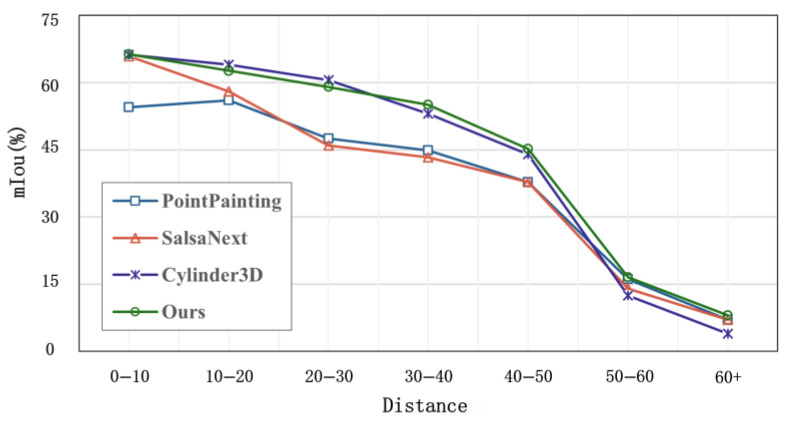
Distance-based semantic evaluation based on the SemanticKITTI dataset. As the distance increases, the point cloud becomes sparse.

In order to evaluate the importance of long-range sensing for autonomous driving safety, we conduct a distance-based evaluation experiment. As shown in Figure 5, the segmentation accuracy of the CMF framework is slightly lower than that of Cylinder3D when the distance is less than 35 m. However, the segmentation accuracy of the CMF framework exceeds that of Cylinder3D when the distance is greater than 35 m.

We attribute this result to the increasing sparsity of the point clouds as the distance increases: LiDAR-only methods degrade in terms of performance, while camera data provides richer information on distant objects. The CMF framework dynamically fuses high-resolution image textures (e.g., vehicle contours) with precise point cloud depth through the use of cross-modal attention mechanisms. In distant sparse regions, image details effectively compensate for sparse LiDAR data. This further confirms that the CMF framework is better suited for semantic segmentation tasks involving sparse LiDAR data.

### 4.5. Ablation Experiments

#### 4.5.1. Impact of Network Components

We analyze the impact on the overall performance of individual components of the CMF network, which include perspective projection (PP), empty space pyramid pooling (ASPP), the simple attention-based residual fusion module (RF), and the cross-attention-based residual fusion module (RCF). The experimental results are detailed in Table 3. To ensure the rigor of the ablation experiments, we use the structurally fine-tuned SalsaNext (spherical projection) as the baseline for our comparison experiments. In regard to unspecified ablation of the network components, the networks involved in the cross-modal feature fusion in the ablation studies use perceptual difference loss sets.

By comparing the experimental results in the above table, we can conclude that although the baseline using perspective projection improves the mIoU by 0.4% compared to the baseline using only spherical projection, the improvement is not significant. However, in the third row, the combination of perspective projection and null pyramid pooling improves the segmentation accuracy by 3% compared to the baseline using spherical projection, proving the effectiveness of this combination in the task of distance image semantic segmentation. Comparing the third line with the fifth line, our proposed CMF network improves the segmentation accuracy by 3.4% with the introduction of camera streams compared to SalsaNext without camera streams. In addition, comparing the fourth row with the fifth row, the cross-attention-based residual fusion module improves the accuracy by 0.8% compared to the simple attention-based residual fusion module. In summary, the experimental results fully demonstrate the importance of introducing camera streams for improving the semantic segmentation accuracy of point clouds and the positive role of the RCF module in the cross-modal feature fusion process.

#### 4.5.2. Effect of the Loss Function

In addition to analyzing the key components in the model, we also thoroughly investigate the impact of different loss function combinations on the model’s performance. Taking the Foc loss and Lov loss as the base loss function groups, we compare the effects of the base, perceptual difference, and perceptual guidance groups on the performance of the CMF network, and validate the facilitating effect of the perceptual guided cross-loss function on cross-modal feature fusion.

According to the experimental results in Table 4, we can see that the CMF framework using the base group in the first row has a segmentation accuracy of only 61.7% based on the validation set. While the CMF using the perceptual difference group in the second row has a segmentation accuracy of up to 63.6% based on the validation set. The CMF using the perceptual guidance group in the third row has a segmentation accuracy of 64.2% based on the validation set, which improves the segmentation accuracy by 2.8% and 0.9% compared with the base group and the perceptual difference group, respectively. The results confirm that the proposed perceptual guided loss significantly improves cross-modal fusion.

**Figure 6:**
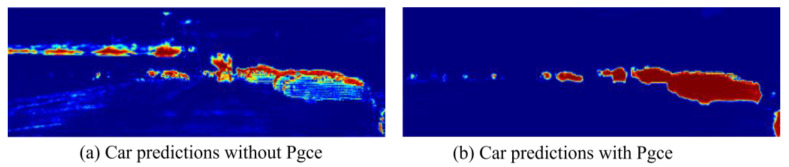
Comparison of prediction results. Networks trained with and without perceptual guided cross-loss function are compared; Pgce denotes perceptual guided cross-loss function. For clarity, only the car-related predictions are shown in red. In the picture, the redder the area is, the greater the possibility that it is predicted to be a car.

To further elaborate the impact of the perceptual guided cross-loss function (Pgce Loss), we present the prediction results of the LiDAR branch with and without Pgce Loss, respectively, in Figure 6. Taking a car as an example, the CMF model with Pgce Loss is able to learn the complete shape of the car during the training process, while the CMF model without introducing the Pgce Loss focuses mainly on local features. This further confirms that the introduction of the Pgce Loss helps the LiDAR branch to better fuse the camera stream information.

### 4.6. Confrontation Analys

**Figure 7:**
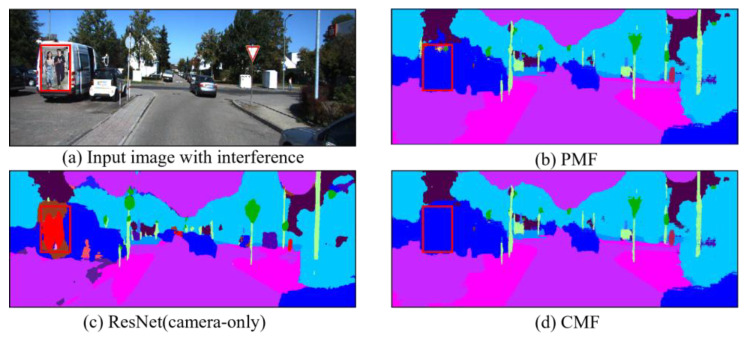
Comparison of CMF with ResNet and PMF in regard to adversarial experiments. ResNet uses only RGB images as inputs, while CMF and PMF use both images and point clouds as inputs. We highlight the inserted interfering objects with red boxes. The red pixels in the prediction results represent people, and the blue pixels represent cars.

When fusing image features, the model is susceptible to information interference, and in order to investigate the robustness of the CMF framework in regard to interfering samples, we insert additional targets into the image (e.g., the poster of the people on the back of the vehicle in Figure 7), while keeping the point clouds unchanged. In addition, based on the SemanticKITTI dataset, we implement a camera stream-only method, ResNet, and a camera and LiDAR fusion method, PMF to predict the results. Note that we do not use any adversarial training techniques during the training process.

As shown in Figure 7c, the methods relying solely on the image stream are prone to interference from distracting objects in the visual data, causing the segmentation results to be biased toward the semantic categories of these distractors. In Figure 7b, most segmentation outputs from PMF (highlighted in the red box) align with the dark blue labels of the “car” category, yet some erroneous segments clearly reflect interference from the distractors. However, the CMF framework addresses this limitation by employing the RCF module during fusion, which deepens the cross-modal feature interaction through the attention mechanism to integrate reliable point cloud features. Additionally, the Pgce Loss function further optimizes the flexibility of the CMF’s feature fusion, reducing the impact of visual noise. Consequently, as demonstrated in Figure 7d, the CMF framework correctly segments the car semantically without misclassifying the human figures in the poster as real entities.

Nevertheless, while the CMF framework demonstrates strong robustness against common distractors (e.g., the poster of humans in Figure 7), we observe some remaining limitations: highly camouflaged distractors (e.g., objects with vehicle-like colors/textures) may still induce misclassifications. In complex dynamic scenarios, the CMF framework exhibits instability in recognizing fast-moving objects due to LiDAR data sparsity and latency. To mitigate these issues, we plan to integrate dynamic filtering methods in future work to enhance the CMF’s adaptability to intricate real-world environments.

### 4.7. Efficiency Analysis

In this section, we evaluate the computational efficiency of the CMF framework based on a GeForce RTX 3090. It is important to note that we optimize the CMF’s efficiency through the use of two key strategies. First, since the camera stream’s predictions are fused into the LiDAR stream, we prune the camera stream’s decoder to accelerate inference. Second, both our PMF and CMF leverage 2D convolutions, enabling straightforward optimization via existing inference toolkits like TensorRT.

In comparison, Cylinder3D relies on 3D sparse convolutions, which are inherently less compatible with TensorRT acceleration. Table 5 reports the inference times of the TensorRT-optimized models. The results demonstrate that the CMF framework achieves state-of-the-art performance based on the SemanticKITTI dataset, while being 3.1 times faster than Cylinder3D (20.1 ms vs. 62.5 ms) with fewer parameters. Compared to PMF, the CMF framework achieves a 1.1× increase in speed (20.1 ms vs. 22.3 ms), alongside an mIoU improvement of 0.9%.

## 5. Conclusions

This study aims to improve the accuracy of semantic segmentation in regard to autonomous driving road scenes by proposing a multi-sensor fusion semantic segmentation method based on cross-modal attention to fully leverage the advantages of point clouds and images. Unlike the commonly used spherical projection method, we employ perspective projection to align point clouds with the camera coordinate system. Additionally, we design a cross-modal residual fusion module with cross-attention at its core, enabling multi-level feature fusion during the feature extraction process, effectively addressing the current issue of insufficient cross-modal feature integration.

Moreover, during model training, we introduce a perceptual guided cross-loss function to further enhance cross-modal feature fusion. A series of experiments conducted based on the SemanticKITTI and nuScenes datasets verify the effectiveness of the cross-modal attention residual fusion module and the perceptual guided cross-loss function. The CMF framework achieved optimal mIoU scores of 64.5% and 79.3% based on the respective datasets. The related interference experiments also demonstrate the superiority and robustness of the CMF framework in regard to the task of semantic segmentation for in-vehicle sensor fusion. Although the CMF framework offers certain improvements in regard to the inference speed and parameter efficiency compared to the PMF, there is still room for further optimization. Additionally, the CMF framework is relatively dependent on precise sensor calibration, and calibration errors in real-world applications may compromise its robustness.

In future research, we plan to address these limitations by exploring lightweight model designs tailored for low-power devices and conducting further experiments to validate the CMF’s adaptability in complex driving environments. Since most cross-modal fusion theories rely on idealized perfect calibration, deep learning-based solutions for automatic sensor alignment/calibration could emerge as a promising direction. Furthermore, we intend to extend the related ideas and techniques in regard to this method to other tasks, such as object detection, to expand its practical applications.

## Figures and Tables

**Table 1:** Semantic KITTI validation set comparison. L denotes the method using only LiDAR. L + C represents the fusion-based method. The black bold numbers indicate the best result.

Method	Input	Car	Bicycle	Motorcycle	Truck	Other Vehicle	Person	Bicyclist	Motorcyclist	Road	Parking	Sidewalk	Other Ground	Building	Fence	Vegetation	Trunk	Terrain	Pole	Traffic Sign	mIoU (%)	Rise/Fall
RandLA-Net [32]	**L**	92.0	8.0	12.8	74.8	46.7	52.3	46.0	0.0	93.4	32.7	73.4	0.1	84.0	43.5	83.7	57.3	73.1	48.0	27.3	50.5	13.7
RangeNet++ [37]	**L**	89.4	26.5	48.4	33.9	26.7	54.8	69.4	0.0	92.9	37.0	69.9	0.0	83.4	51.0	83.3	54.0	68.1	49.8	34.0	51.2	13.0
SequeeneSegV3 [53]	**L**	87.1	34.3	48.6	47.5	47.1	58.1	53.8	0.0	95.3	43.1	78.2	0.3	78.9	53.2	82.3	55.5	70.4	46.3	33.2	53.3	10.9
SalsaNext [38]	**L**	90.5	44.6	49.6	86.3	54.6	74.0	81.4	0.0	93.4	40.6	69.1	0.0	84.6	53.0	83.6	64.3	64.2	54.4	39.8	59.4	4.8
SPVNAS [54]	**L**	96.5	44.8	63.1	59.9	64.3	72.0	86.0	0.0	93.9	42.4	75.9	0.0	88.8	59.1	88.0	67.5	73.0	63.5	44.3	62.3	1.9
Cylinder3D [33]	**L**	96.4	61.5	78.2	66.3	69.8	80.8	93.3	0.0	94.9	41.5	78.0	1.4	87.5	50.0	86.7	72.2	68.8	63.0	42.1	**64.9**	−0.7
PointPainting [46]	**L + C**	94.7	17.7	35.0	28.8	55.0	59.4	63.6	0.0	95.3	39.9	77.6	0.4	87.5	55.1	87.7	67.0	72.9	61.8	36.5	54.5	9.7
RGBAL [45]	**L + C**	87.3	36.1	26.4	64.6	54.6	58.1	72.7	0.0	95.1	45.6	77.5	0.8	78.9	53.4	84.3	61.7	72.9	56.1	41.5	56.2	8.0
PMF [47]	**L + C**	94.6	47.0	62.1	66.4	74.2	78.1	70.7	0.0	96.2	42.4	80.5	0.1	86.5	59.2	88.5	72.7	75.1	63.7	42.0	63.2	1.0
CMF (Ours)	**L + C**	**96.6**	45.6	66.0	61.6	**76.4**	**78.8**	75.5	**0.5**	**96.7**	**45.2**	**80.9**	**1.5**	**89.5**	**64.3**	**88.9**	**72.9**	75.4	**65.9**	**43.5**	64.5	

**Table 2:** Comparisons based on the nuScenes validation set. L denotes the method using only LiDAR. L + C represents the fusion-based method. The black bold numbers indicate the best result.

Method	Input	Barrier	Bicycle	Bus	Car	Construction	Motorcycle	Pedestrian	Traffic Cone	Trailer	Truck	Drivable	Other Flat	Sidewalk	Terrain	Manmade	Vegetation	mIoU (%)	Rise/Fall
RangeNet++ [37]	**L**	66.0	21.3	77.2	80.9	30.2	66.8	69.6	52.1	54.2	72.3	94.1	66.6	63.5	70.1	83.1	79.8	65.5	13.8
PolarNet [55]	**L**	74.7	28.2	85.3	90.9	35.1	77.5	71.3	58.8	57.4	76.1	96.5	71.1	74.7	74	87.3	85.7	71.0	8.3
SalsaNext [38]	**L**	74.8	34.1	85.9	88.4	42.2	72.4	72.2	63.1	61.3	76.5	96	70.8	71.2	71.5	86.7	84.4	72.2	7.1
RangeFormer [39]	**L**	78	45.2	94	92.9	58.7	83.9	77.9	69.1	63.7	85.6	96.7	74.5	75.1	75.3	89.1	87.5	78.1	1.2
2DPASS [16]	**L** **+ C**	74.4	44.3	93.6	92	54	79.7	78.9	57.2	72.5	85.7	96.2	72.7	74.1	74.5	87.5	85.4	76.4	2.9
2D3DNet [17]	**L + C**	78.3	55.1	95.4	87.7	59.4	79.3	80.7	70.2	68.2	86.6	96.1	74.9	75.7	75.1	91.4	89.9	79.0	0.3
PMF [47]	**L + C**	74.1	46.6	89.8	92.1	57	77.7	80.9	70.9	64.6	82.9	95.5	73.3	73.6	74.8	89.4	87.7	76.9	2.4
CMF (Ours)	**L + C**	**78.4**	54.5	**95.7**	91.1	**64.2**	**85.2**	79.6	**73.2**	66.7	85.5	95.9	74.3	75	74.9	88.9	86.8	**79.3**	

**Table 3:** Ablation study of network components based on the SemanticKITTI validation set. The symbol “✓” represents the use of this module.

	Baseline	PP	ASPP	RF	RCF	mIoU (%)
1	✓					57.2
2	✓	✓				57.6
3	✓	✓	✓			60.2
4	✓	✓	✓	✓		62.8
5	✓	✓	✓		✓	63.6

**Table 4:** CMF ablation study of loss function sets based on SemanticKITTI validation set. The symbol “✓” represents the use of the corresponding loss function.

	Foc Loss + Lov Loss	Pe Loss	Pgce Loss	mIoU (%)
1	✓			61.7
2	✓	✓		63.6
3	✓		✓	64.5

**Table 5:** Inference time of different methods based on SemanticKITTI dataset using TensorRT.

	#FLOPs	#Params.	Inference Time	mIoU
PointPainting	51.0 G	28.1 M	2.3 ms	54.5%
RGBAL	55.0 G	13.2 M	2.7 ms	56.2%
SalsaNext	31.4 G	6.7 M	1.6 ms	59.4%
Cylinder3D	-	55.9 M	62.5 ms	64.9%
PMF	854.7 G	36.3 M	22.3 ms	63.6%
CMF (ours)	620.2 G	34.7 M	20.1 ms	64.5%

## Data Availability

The data that support the findings of this study were derived from the following resources available in the public domain: [University of Bonn, www.semantic-kitti.org (accessed on 1 March 2025)].
